# Long non-coding RNA LOXL1-AS1: a potential biomarker and therapeutic target in human malignant tumors

**DOI:** 10.1007/s10238-024-01355-7

**Published:** 2024-05-02

**Authors:** Xiao-Ping Fu, Chun-Yan Ji, Wen-Qian Tang, Ting-Ting Yu, Lei Luo

**Affiliations:** 1 Department of Health Management Center, Hubei Provincial Hospital of Traditional Chinese Medicine, Hongshan District, 856 Luoyu Road, Wuhan, 430070 People’s Republic of China; 2 Department of Gastroenterology, Hubei Provincial Hospital of Traditional Chinese and Western Medicine, Wuhan, 430015 People’s Republic of China; 3 School of Clinical Medical, Hubei University of Chinese Medicine, Wuhan, 443000 People’s Republic of China

**Keywords:** Long non-coding RNA, LOXL1-AS1, Cancer, Biomarker, Therapeutic target

## Abstract

Long non-coding RNAs (lncRNAs) are transcripts that contain more than 200 nucleotides. Despite their inability to code proteins, multiple studies have identified their important role in human cancer through different mechanisms. LncRNA lysyl oxidase like 1 antisense RNA 1 (LOXL1-AS1), a newly discovered lncRNA located on human chromosome 15q24.1, has recently been shown to be involved in the occurrence and progression of various malignancies, such as colorectal cancer, gastric cancer, hepatocellular carcinoma, prostate cancer, non-small cell lung cancer, ovarian cancer, cervical cancer, breast cancer, glioma, thymic carcinoma, pancreatic carcinoma. LOXL1-AS1 acts as competitive endogenous RNA (ceRNA) and via sponging various miRNAs, including miR-374b-5p, miR-21, miR-423-5p, miR-589-5p, miR-28-5p, miR-324-3p, miR-708-5p, miR-143-3p, miR-18b-5p, miR-761, miR-525-5p, miR-541-3p, miR-let-7a-5p, miR-3128, miR-3614-5p, miR-377-3p and miR-1224-5p to promote tumor cell proliferation, invasion, migration, apoptosis, cell cycle, and epithelial–mesenchymal transformation (EMT). In addition, LOXL1-AS1 is involved in the regulation of P13K/AKT and MAPK signaling pathways. This article reviews the current understanding of the biological function and clinical significance of LOXL1-AS1 in human cancers. These findings suggest that LOXL1-AS1 may be both a reliable biomarker and a potential therapeutic target for cancers.

## Introduction

Cancer is a serious threat to human health all over the world due to its concealment and recurrence and the difficulty of early diagnosis affects the prognosis of patients [1, 2]. According to statistics, the number of new cancer cases and deaths in the world in 2020 were 19.29 million and 9.96 million, respectively, while the number of new cancer cases and deaths in China were 4.57 million and 3 million, respectively [3]. In recent years, with the increase in cancer cell resistance to chemotherapeutic drugs and the decrease in sensitivity to radiotherapy, the clinical efficacy of traditional radiotherapy and chemotherapy is limited. Therefore, in-depth study of the pathogenesis of cancer and looking for new and effective targets for cancer treatment are of great significance for early diagnosis of cancer and improving the overall survival rate of patients.

Non-coding RNAs (NcRNAs) are functional RNA molecules that are not translated into proteins [4]. Although ncRNAs lacks the potential to encode proteins, they can affect the expression of many molecular targets and drive specific cellular biological responses and destinies. Studies have shown that ncRNAs regulates gene expression at many levels, including replication, transcription and post-transcription, and participates in chromatin modification, cell differentiation, protein function regulation and disease development [5]. Through high-throughput RNA sequencing, there has been a rapid and accurate identification of tens of thousands of ncRNAs in organisms ranging from bacteria to humans. NcRNAs are broadly classified into microRNAs (miRNAs), long non-coding RNAs (lncRNAs), and circular RNAs (circRNAs). MiRNAs is an 18-25nt ncRNA molecule, which usually interacts with promoter region, 3' UTR and 5' UTR region, coding sequence and gene promoter to inhibit translation and/or promote mRNA degradation. Therefore, inhibiting the expression of target molecules may play an important role in biological metabolism, tumorigenesis and immune inflammation [6]. CircRNAs is a covalently closed ring molecule with no 5' or 3' end, which is formed by the "reverse splicing" event. As a stable RNA molecule in a variety of cells and tissues, circRNAs has been proved to play a role in many diseases through the sponge action of miRNA, the regulation of transcription and splicing of target genes, protein interaction and epigenetic regulation [7]. Some specific ncRNAs are reported to be lost in genetic diseases like Prader–Willi syndrome and expression levels of some ncRNAs are associated with clinicopathological features in cancerous patients and the occurrence and progression of ischemic stroke [8, 9].

LncRNAs are a large and highly diverse class of ncRNAs that are > 200 nucleotides in length [10]. Initially, lncRNAs were regarded as nonfunctional gene transcription noise, so it received limited attention [11]. However, further studies have found that LncRNAs have multiple functions because their functional libraries depend on their cellular localization and complex interactions with DNA, RNA and proteins. Using these characteristics, lncRNAs can control chromatin kinetics, regulate the assembly and operation of non-membrane-bound nucleosomes, change the stability and translation efficiency of cytoplasmic mRNA, and interfere with signal transduction pathway [12–14]. It is worth noting that these multifaceted mechanisms jointly promote the regulation of gene expression patterns in various biological and pathophysiological backgrounds, including cancer.

**Fig. 1:**
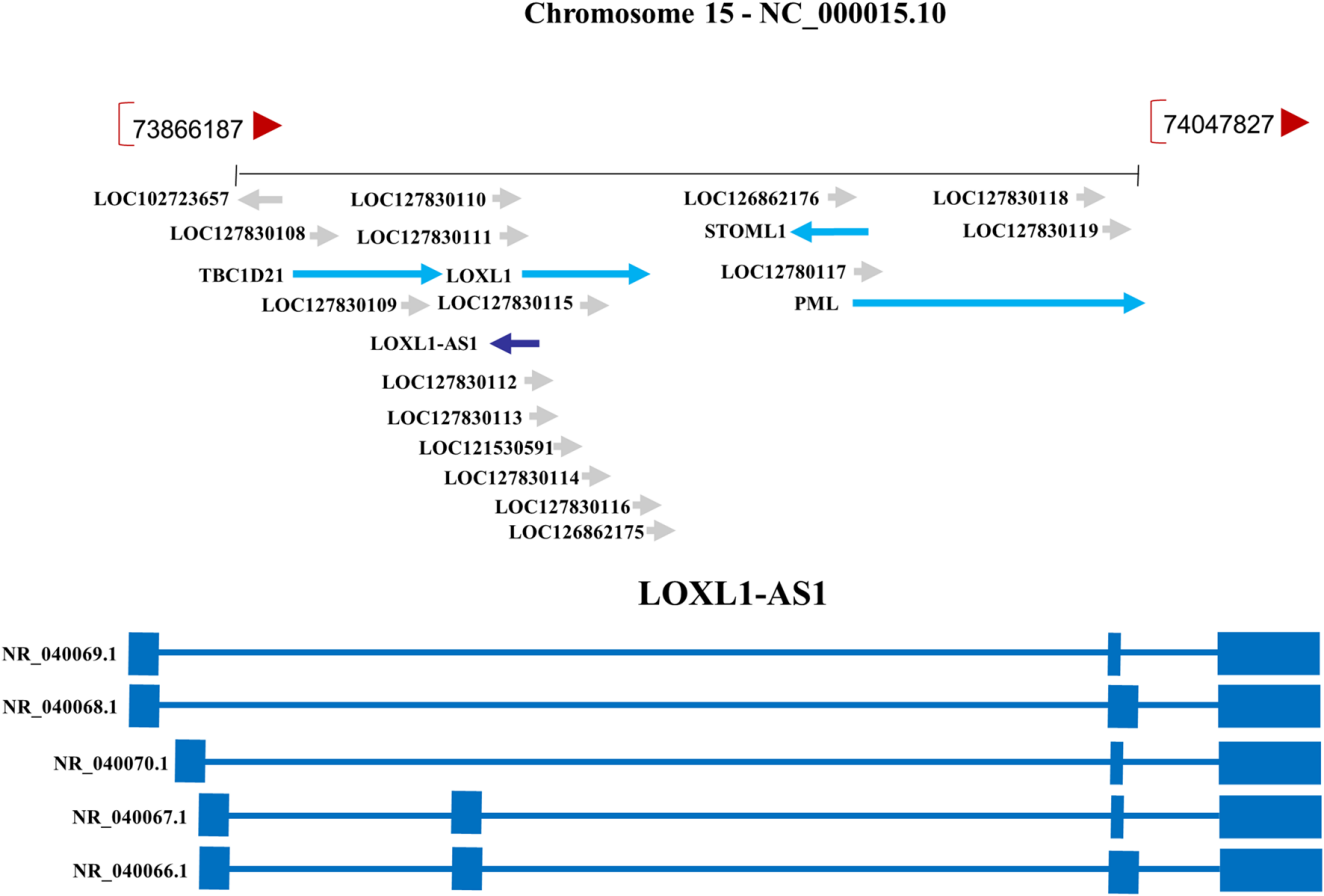
Schematic diagram of the formation of lncRNA LOXL1-AS1. The LOXL1-AS1 gene is located on chromosome 15q24.1 and contains five transcripts: NR_040069.1, NR_04068.1, NR_040070.1, NR_040067.1, and NR_040066.1

**Fig. 2:**
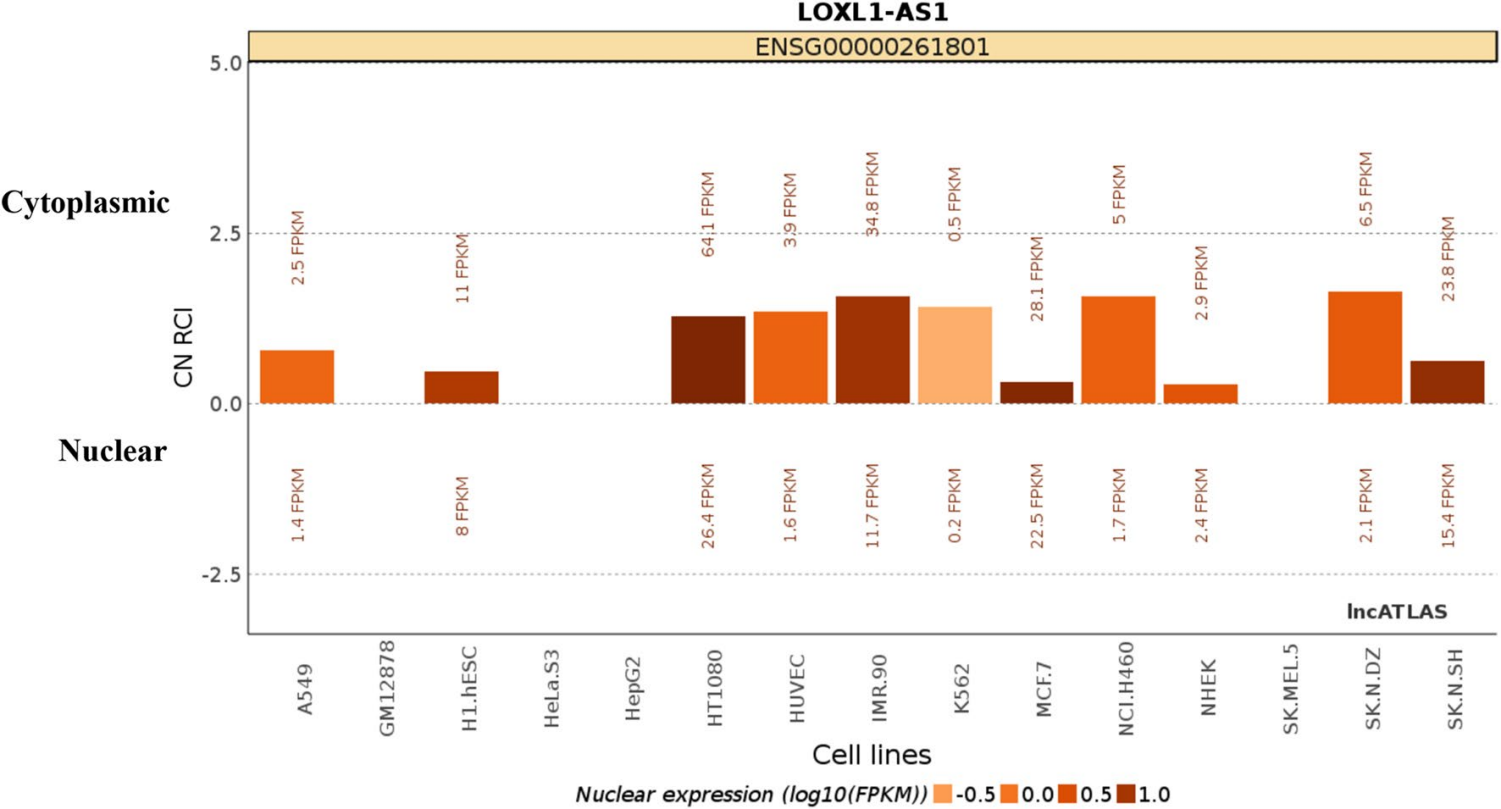
The localization of LOXL1-AS1 in common cell lines is mainly located in the cytoplasm (bar plot from lncATLAS analysis; http://lncatlas.crg.eu/)

LncRNA lysyl oxidase like 1 antisense RNA 1 (LOXL1-AS1) is a new type of lncRNA recently discovered by sequencing and genetic analysis. The NCBI database (https://www.ncbi.nlm.nih.gov/) shows that LOXL1-AS1 is located on human chromosome 15q24.1 and contains 5 transcripts (Fig. 1). In addition, we found that LOXL1-AS1 was mainly localized in the cytoplasm through lncATLAS analysis (http://lncatlas.crg.eu/) (Fig. 2). It is encoded in the pair strand of lysyl oxidase-like 1 (LOXL1) gene and has been proved to be carcinogenic in many human cancers. As a carcinogenic factor, LOXL1-AS1 plays a powerful regulatory role in tumor progression by interacting with a variety of signal molecules to affect the biological characteristics of various cancer cells, such as changing their invasion, proliferation and drug resistance [15–17]. LOXL1-AS1 may also affect non-cancer diseases such as periodontitis and osteoarthritis [18, 19].

In the article, we review the function and mechanism of LOXL1-AS1 in a variety of malignant tumors (Table 1) and emphasize its potential clinical value (Table 2). Our research shows that LOXL1-AS1 may be a potential cancer biomarker or therapeutic target, which provides guidance and reference for follow-up researchers to further clarify its role in cancer regulation and develop related clinical targeted drugs.

**Table 1:** Functions and mechanisms of LOXL1-AS1 in various cancers

Cancer type	Cell lines	Expression	Targets	Related biological activities	Reference
Endometrial cancer	HAC-1A, KLE, Ishikawa, RL-95–2	Upregulation	miR-28-5p/RAP1B	Proliferation↑, invasion↑, migration↑ and apoptosis↓ in vitro, tumor size↑ in vivo	[17]
Colorectal cancer	HCT8, LoVo, SW620, Caco2, SW1463	Upregulation	miR-708-5p/CD44-EGFR	Proliferation↑, migration↑, invasion↑	[22]
Colorectal cancer	Caco2, SW620, HCT-116, SW480	Upregulation	miR-1224-5p/miR‑761/HK2	Proliferation↑, invasion↑, migration↑, glycolysis↑ and apoptosis↓ in vitro, tumor size↑ and weight↑ in vivo	[23]
Gastric cancer	MKN‐45, AGS, SGC7901, MGC-803	Upregulation	miR‐708‐5p/USF1	Proliferation↑, migration↑, epithelial-mesenchymal transition↑ and stemness↑ in vitro, tumor size↑ and weight↑ in vivo	[27]
Hepatocellular carcinoma	HCCLM3, Huh-7, SK-HEP-1	Upregulation	miR-3614-5p/YY1	Proliferation↑, invasion↑, migration↑, apoptosis↓	[35]
Liver cancer	SK‑HEP‑1, Hep3B, SNU1	Upregulation	miR‑377‑3p/NFIB	Glucose metabolism↑, proliferation↑, migration↑ and epithelial–mesenchymal transition↑	[36]
Prostate cancer	PC3, DU145	Upregulation	miR-541-3p/CCND1	Proliferation↑ and cell cycle↑	[38]
Prostate cancer	DU-145	Upregulation	miR-let-7a-5p/EGFR	Proliferation↑, migration↑ and apoptosis↓ in vitro, tumor size and weight↑ in vivo	[39]
Non-small cell lung cancer	H1299, A549, H520, H596	Upregulation	miR-3128/RHOXF2	Proliferation↑, invasion↑ and migration↑	[42]
Non-small cell lung cancer	H23、A549、H1299, SPC-A1	Upregulation	miR-324-3p	Proliferation↑ and invasion↑	[43]
Lung adenocarcinoma	H1299, H1975, A549, SPC‐A1	Upregulation	miR‐423‐5p/MYBL2	Proliferation↑, migration↑, apoptosis↓	[44]
Ovarian cancer	SKOV-3	Upregulation	miR-761	Proliferation↑, apoptosis↓	[48]
Ovarian cancer	A2780, SKOV3, Caov-3, OVCAR3	Upregulation	miR‐18b‐5p/VMA21	Growth↑, invasion↑, migration↑	[49]
Cervical cancer	SiHa	Downregulation	miR-21/RHOB	Invasion↓ and migration↓	[52]
Cervical cancer	HeLa, SiHa, CaSki, ME-180	Upregulation	miR-423-5p/ENC1/MEK/ERK	Proliferation↑ and migration↑ in vitro, tumor growth↑ and tumor size↑ in vivo	[53]
Breast cancer	T47D, MCF7, MDA-MB-231, MDA-MB-468	Upregulation	miR-708-5p/EZH2	Invasion↑ and migration↑ in vitro, metastasis↑ in vivo	[55]
Breast cancer	MCF-7, MDA-MB-231, BT549, SKBR-3	Upregulation	miR-143-3p	Proliferation↓, invasion↓, migration↓, apoptosis↑	[56]
Glioma	U87MG	Upregulation	NF-kB	Proliferation↑	[59]
Glioma	HEK293T, U87, U251	Upregulation	miR-374b-5p/MMP14	Proliferation↑, migration↑, invasion↑ and vasculogenic mimicry↑ in vitro, tumor size↑ and survival time↓ in vivo	[63]
Osteosarcoma	MG63、U2OS、Sao	Upregulation	PI3K/AKT pathway	Proliferation↑, migration↑, invasion↑	[64]
Cholangiocarcinoma	RBE, HuCCT1, QBC939, Huh-28, CCLP1	Upregulation	miR-324-3p/ATP-ABCA1	Proliferation↑, invasion↑, migration↑, apoptosis↓	[65]
Laryngocarcinoma	Tu-177, M4E, NU-899, SNU-46, AMC-HN-8	Upregulation	miR-589-5p/TRAF6	Proliferation↑, migration↑ and epithelial-mesenchymal transition↑ in vitro, tumour growth↑ in vivo	[66]
Medulloblastoma	Daoy, D283, D425, D341, D458	Upregulation	PI3K/AKT pathway	Proliferation↑, metastasis↑ and apoptosis↓ in vitro, tumor size↑ and weight↑ in vivo	[67]
Retinoblastoma	Y79, WERI-Rb1	Upregulation	MAPK pathway	Proliferation↑, invasion↑ and apoptosis↓ in vitro, tumor size↑ and weight↑ in vivo	[68]
Renal cell carcinoma	786-O, A-498, 769-P	Upregulation	miR-589-5p/CBX5	Proliferation↑, migration↑	[69]
Esophageal squamous cell carcinoma	KYSE30, EC109	Upregulation	DESC1	Proliferation↑, invasion↑, migration↑, apoptosis↓	[70]
Thymic carcinoma	Thy0517, Ty-82	Upregulation	miR‑525‑5p/HSPA9	Growth↑, invasion↑ and apoptosis↓ in vitro, tumour growth↑ in vivo	[71]
Pancreatic cancer	SW1990, BXPC-3, PANC-1, PaCa-2	Upregulation	miR-28-5p/SEMA7A	Proliferation↑, migration↑	[72]

**Table 2:** The expression and clinical characteristics of LOXL1-AS1 in human cancers

Cancer type	Numbers of clinical samples	Expression	Clinical characteristics	Prognostic implication of LOXL1-AS1 overexpression	Property	Reference
Endometrial cancer	50	Upregulation	Histological grade, FIGO stage and lymphatic metastasis	Poor	Oncogene	[17]
Colorectal Cancer	40	Upregulation	Tumor size, differentiation, TNM stage, liver metastasis, and MSI stage	Poor	Oncogene	[22]
Colorectal Cancer	27	Upregulation	TNM grade, lymph node metastasis and tumor size	Poor	Oncogene	[23]
Gastric cancer	84	Upregulation	Poor prognosis	Poor	Oncogene	[27]
Liver cancer	38	Upregulation	Overall survival	Poor	Oncogene	[36]
Ovarian cancer	185	Upregulation	FIGO stage, distant metastasis and overall survival	Poor	Oncogene	[47]
Ovarian cancer	45	Upregulation	Overall survival	Poor	Oncogene	[49]
Cervical cancer	40	Upregulation	Lymph node metastasis, TNM stage and distant metastasis	Poor	Oncogene	[53]
Breast Cancer	85	Upregulation	TNM grade, lymph node metastasis	Poor	Oncogene	[55]
Glioma	169	Upregulation	Overall survival	Poor	Oncogene	[59]
Osteosarcoma	58	Upregulation	Enneking stage, tumor size, distant metastasis, histological grade, and overall survival	Poor	Oncogene	[64]
Cholangiocarcinoma	64	Upregulation	Lymph node invasion, TNM stages and poor prognosis	Poor	Oncogene	[65]
Esophageal squamous cell carcinoma	45	Upregulation	Lymph node metastasis	Poor	Oncogene	[70]
Thymic carcinoma	70	Upregulation	Poor prognosis	Poor	Oncogene	[71]

## Role of LOXL1-AS1 in various cancers

### Colorectal cancer

Colorectal cancer (CRC) is a malignant tumor with high morbidity and mortality, data show that new CRC patients account for about 10% of all cancer patients and CRC deaths account for about 9% of global cancer-related deaths each year [3, 20]. Although a variety of treatments, including endoscopic and surgical resection, radiotherapy, local ablation, chemotherapy, targeted therapy and systemic therapy, have been combined to improve the prognosis of patients with CRC, the overall survival rate of patients is still not satisfactory [21]. Therefore, in-depth study of its pathophysiological mechanism to determine the new diagnosis and prognosis of CRC is very necessary.

Quantitative Real-Time PCR (qRT-PCR) analysis revealed that the level of LOXL1-AS1 in CRC tissues was significantly higher than that in adjacent normal tissues, and the expression level was positively correlated with tumor size, differentiation, tumor node metastasis (TNM) stage, liver metastasis and microsatellite instability (MSI) stage. The same conclusion was reached in a variety of CRC cell lines. Further studies have revealed that LOXL1-AS1 enhances the proliferation, migration, invasion and progression of CRC through sponge miR-708-5p regulating CD44-EGFR signal pathway [22]. Guo et al. [23] found that knocking down LOXL1-AS1 inhibits the CRC cells proliferation, migration, invasion and glycolysis and induces apoptosis in vitro. Silencing LOXL1-AS1 can inhibit CRC tumor growth in vivo. The above studies suggest that LOXL1-AS1 may be a promising therapeutic target for CRC.

### Gastric cancer

Gastric cancer (GC) is a cancer with a high incidence worldwide. About 800,000 people die of GC every year, and more than 70% of the diagnosed GC patients come from developing countries [24, 25]. Because most patients with GC are diagnosed at an advanced stage, resulting in a high mortality rate [26]. Therefore, exploring new GC treatment strategies is of great significance to improve the early diagnosis and overall survival rate of GC patients. Sun et al. [27] found that the expression of LOXL1-AS1 was significantly increased in 84 GC patients and four kinds of human GC cell lines, and the high expression of LOXL1-AS1 was related to the poor prognosis of the patients. In addition, in vivo confirmed that LOXL1-AS1 can promote the progression of GC in mouse models. Upstream stimulating factor 1 (USF1) is an important regulatory factor in many diseases, including cancer, and its expression is significantly increased in GC tissues and cells [28]. Besides, LOXL1-AS1 promotes the progress of GC by upregulating the expression of USF1 targeting miR-708-5p [27]. Knocking down the expression of LOXL1-AS1 inhibit the invasion and migration and inhibit the epithelial-mesenchymal transformation (EMT) of GC cells by regulating the expression of PIK3CA through miR-142-5p [29]. These results suggest that the LOXL1-AS1 may be a novel therapeutic target for CRC.

### Hepatocellular carcinoma

Hepatocellular carcinoma (HCC) is one of the most common malignant tumors in the world, accounting for about 70% of liver malignant tumors [30]. Data show that HCC is the third leading cause of cancer-related death with a 5-year survival rate of only 18% [31, 32]. HCC has the characteristics of high recurrence rate and poor prognosis, which is partly due to its invasiveness and metastasis [33]. Therefore, there is an urgent need for a reliable HCC progression biomarker to improve clinical strategy. Liu et al. [34] reported that LOXL1-AS1 was generally increased in HCC tissues and cells, while downregulation of LOXL1-AS1 significantly inhibited cell proliferation, migration and invasion. Bioinformatics showed that miR-3614-5p could be absorbed by LOXL1-AS1 sponge, while Yin Yang 1 (YY1) was confirmed to be the target gene of miR-3614-5p, and YY1 deletion could inhibit the malignant behavior of HCC cells. To sum up, LOXL1-AS1 up-regulates YY1 through sponge miR-3614-5p to promote the proliferation, migration, invasion and inhibit apoptosis of HCC cells [35]. Kaplan–Meier analysis showed that high expression of LOXL1-AS1 was associated with lower overall survival rate in patients with HCC [36]. Taken together, these findings confirm that LOXL1-AS1 disorders play a key role in HCC, but further studies are needed to determine its clinical importance.

### Prostate cancer

Prostate cancer** (**PCa) is one of the most common malignant tumors in men and a global problem that threatens men's health. In 2022, there were more than 268,490 new PCa cases and 34,500 deaths in the USA, 80% of PCa patients have carcinoma in situ, another 20% have metastasis and the 5-year survival rate of patients with metastatic PCa is only about 30% [37]. The expression of LOXL1-AS1 increased in PCa cells, while knocking down LOXL1-AS1 significantly inhibited the proliferation of PCa cells and inhibited the cell cycle progression by increasing the percentage of cells in G0/G1 phase and decreasing the percentage of cells in S phase. Further experiments showed that LOXL1-AS1 regulated the expression of miR541-3p, and then regulated the expression of cell cycle regulator CCND1 (Cyclin D1), thus regulating the proliferation and cell cycle progression of PCa cells [38]. Chemotherapy plays an important role in preventing tumor recurrence and metastasis. Unfortunately, drug resistance prevents cancer patients from being completely cured. Doxorubicin is one of the main drugs in the treatment of PC, the increase of drug resistance after repeated administration leads to the decrease of efficacy [39]. The expression level of LOXL1-AS1 in doxorubicin-resistant PCa cells was downregulated and upregulation of LOXL1-AS1 can promote cell proliferation, migration and inhibit apoptosis through the LOXL1-AS1/miR-let-7a-5p/EGFR axis achieved, which may become a new strategy for the treatment of drug-resistant PCa patients [40]. To sum up, LOXL1-AS1 promotes the progress of Pca and may be used for treatment, which needs to be explored by follow-up experiments in vivo.

### Non-small cell lung cancer

As a malignant tumor with a high incidence in the world, lung cancer causes the most deaths among all malignant tumors with about 1.8 million patients dying from lung cancer every year [3]. Non-small cell lung cancer (NSCLC) is one of the main types of lung cancer, accounting for about 85% [41]. LOXL1-AS1 is involved in the occurrence and development of NSCLC, and the expression level of LOXL1-AS1 is significantly increased in 43 NSCLC tissues, and the expression level is closely related to TNM stage and metastasis. In addition, the expression level of LOXL1-AS1 in the four NSCLC cell lines was higher than that in normal lung epithelial cells [42]. Xie et al. [43] reached the same conclusion and overexpression of LOXL1-AS1 could regulate the expression of miR-324-3p to promote the proliferation and invasion of NSCLC cells. As one of the main subtypes of NSCLC, lung adenocarcinoma (LUAD) is characterized by high morbidity and mortality. The expression of LOXL1-AS1 was also significantly increased in LUAD tissues and cells, and knocking down LOXL1-AS1 decreased cell proliferation and migration, and promoted cell apoptosis. It was identified that LOXL1-AS1 is mainly located in the cytoplasm and can act as a sponge of miR-423-5p, regulate the expression of MYBL2 and promote the progression of LUAD [44]. In summary, LOXL1-AS1 plays a role in promoting the malignant progression of NSCLC and may be a therapeutic target for NSCLC.

### Ovarian cancer

Ovarian cancer (OC) is one of the most common malignancies in women and the most deadly gynecological malignancies [45]. The 5-year survival rate for OC has remained at about 47% for nearly two decades with no significant improvement [46]. Because of the high recurrence rate of OC, early diagnosis and effective treatment have become important research targets. The expression level of LOXL1-AS1 in OC patients was significantly higher than that in healthy controls and the expression level of LOXL1-AS1 was correlated with advanced FIGO stage and distant metastasis. Kaplan–Meier method was used to analyze the relationship between circulating LOXL1-AS1 and overall survival, and the results showed that OC patients with higher circulating LOXL1-AS1 levels may have poorer overall survival [47]. Similarly, the expression of LOXL1-AS1 in OC cells was significantly higher than that in normal ovarian cells, down-regulation of LOXL1-AS1 inhibited the proliferation and promoted apoptosis of OC cells and the mechanism was related to the targeted regulation of miR-761 [48]. Potential downstream targets of LOXL1-AS1 were screened through bioinformatics analysis, and miR-18b-5p/Vacuolar ATPase Assembly Factor VMA21 (VMA21) was confirmed as the direct target gene. Finally, the authors evaluated the relationship between LOXL1-AS1, miR-18b-5p, and VMA21 using OC cells. Results showed that the expression of LOXL1-AS1 was positively correlated with VMA21 and negatively correlated with miR-18b-5p. Together, these data suggest that LOXL1-AS1 promotes the growth, migration, and invasion of OC cells through the miR-18b-5p/VMA21 axis [49]. Collectively, they concluded that LOXL1-AS1 is a potential carcinogenic lncRNA in OC, making it a promising target for OC therapy.

### Cervical cancer

Cervical cancer (CC) is the fourth leading cause of death among women. Despite the existence of well-established CC screening methods and the development of human papillomavirus vaccines, CC remains the leading cause of cancer-related deaths in low- and middle-income countries [50]. Cervical squamous cell carcinoma (CSCC) is the most common subtype of CC, accounting for approximately 90% of all CC cases [51]. By analyzing the TCGA data, the expression of LOXL1-AS1 in CSCC tissues was significantly lower than that in non-tumor tissues. The expression of LOXL1-AS1 in CSCC patients was also downregulated by qRT-PCR. RNA immunoprecipitation and luciferase reporter genes confirmed that miR-21 interacts with LOXL1-AS1, and RHOB is the target of miR-21. LOXL1-AS1 inhibits the invasion and migration of CSCC cells by regulating the miR-21/RHOB axis [52]. Interestingly, another report showed that the expression of LOXL1-AS1 was up-regulated in both CC tissues and cells, and decreased LOXL1-AS1 inhibited the proliferation and migration of CC cells, as well as the growth and metastasis of tumors [53]. The difference between the two studies may be due to the different pathological types of CC. Therefore, the role of LOXL1-AS1 in CC remains to be further determined.

### Breast cancer

Breast cancer (BC) is the most common malignancy in women, about 2.1 million people in the United States are diagnosed with BC every year [54]. Early diagnosis is the key to improve the prognosis of breast cancer. Therefore, searching for early diagnostic markers is of great significance for early diagnosis. Analysis of 42 cancer-related lncRNAs in MCF7 (with low metastatic potential) and MDA-MB-231 (with high metastatic potential) cells showed that the expression of LOXL1-AS1 was significantly increased in MDA-MB-231 cells [55]. Meanwhile, the expression of LOXL1-AS1 was significantly increased in BC tissues and the elevated level was correlated with tumor stage and lymph node metastasis. Overexpression of LOXL1-AS1 enhanced the migration and invasion ability of BC cells. Luciferase reporter gene analysis showed that LOXL1-AS1 targets miR-143-3p in BC cells, thereby regulating the proliferation, migration and invasion of BC cells [56]. To sum up, these evidence suggest that LOXL1-AS1 plays a carcinogenic role in BC prognosis and progression.

### Glioma

Glioma is the most common malignant tumor originating in the central nervous system and is characterized by high mortality and low survival [57]. Due to glioma is a complex disease with multiple molecular regulations, current treatment options are insufficient to reduce mortality and improve patient life expectancy. Therefore, it is critical to investigate new prognostic indicators for glioma and identify other targets for treating this deadly disease. Studies have shown that nuclear factor κB (NF-κB) signaling pathway plays an important role in the development of glioma by mediating inflammation and immune response [58]. Silencing LOXL1-AS1 inhibit glioma cell proliferation and participate in regulating the NF-κB signaling pathway by inhibiting RELB directly [59]. Vasculogenic mimicry (VM), as a newly discovered form of angiogenesis that promotes angiogenesis in malignant tumors, has been widely reported in a variety of tumors [60, 61]. Tia1-associated protein (TIAR) can prolong the survival of astrocytoma and glioblastoma multiforme patients [62]. The expression of LOXL1-AS1 is high and TIAR is low in glioma cells and tissues. On the mechanism, TIAR reduces the stability of LOXL1-AS1, while LOXL1-AS1 sponge miR-374-5p up-regulates MMP14, promoting the proliferation, migration, invasion and VM of glioma cells [63]. Therefore, these studies provide new possibilities for diagnosis and treatment strategies for glioma patients, but further research is needed.

### Other cancers

The expression level of LOXL1-AS1 in osteosarcoma (OS) cells and tissues is higher than that in normal bone cells and bone tissues [64]. In addition, the expression level of LOXL1-AS1 was closely correlated with Enneking stage, tumor size, distant metastasis, histological grade and overall survival time of OS patients. Kaplan–Meier survival analysis showed that high expression of LOXL1-AS1 in OS was associated with shorter overall survival. Studies on loss of function have shown that knocking down LOXL1-AS1 significantly inhibit the proliferation, migration and invasion of OS cells by inhibiting the PI3K-AKT pathway [64]. High-throughput sequencing technology screening showed that LOXL1-AS1 was overexpressed in cholangiocarcinoma (CCA). Further luciferase reporting and rescue experiments showed that LOXL1-AS1, acting as ceRNA, increased the level of ATP-binding cassette transporter A1 (ABCA1) by sponging miR-324-3p, promoted the proliferation, migration and invasion of CCA cells, and inhibited apoptosis, thus playing a carcinogenic role in CCA [65]. Laryngocarcinoma is a common malignant tumor of the upper respiratory tract, and the expression of LOXL1-AS1 is significantly increased in laryngocarcinoma cells, and promotes the proliferation, migration and EMT of laryngocarcinoma cells [66]. In human medulloblastoma xenotransplantation model, knocking down LOXL1-AS1 can significantly inhibit tumor growth and promote tumor cell apoptosis. In addition, knocking down LOXL1-AS1 inhibits cell migration and reverses EMT [67]. In retinoblastoma (RB), LOXL1-AS1 can also act as an oncogene to regulate MAPK signaling pathway, promote RB proliferation and invasion and inhibit cell apoptosis [68]. LOXL1-AS1 is also highly expressed in renal cell carcinoma (RCC) [69], esophageal squamous cell carcinoma (ESCC) [70], thymic carcinoma (TC) [71], pancreatic carcinoma (PC) [72] and endometrial carcinoma (EC) [17], promoting tumor progression. In summary, these studies reveal that LOXL1-AS1 has carcinogenic effects on a variety of malignant tumors, but more in-depth research is needed.

### Regulatory mechanisms of LOXL1-AS1 in cancers

The mechanisms of LOXL1-AS1 involved in cancer can be summarized into three types: ceRNA function, protein interaction and pathway interaction.

### Interaction with miRNAs

**Fig. 3:**
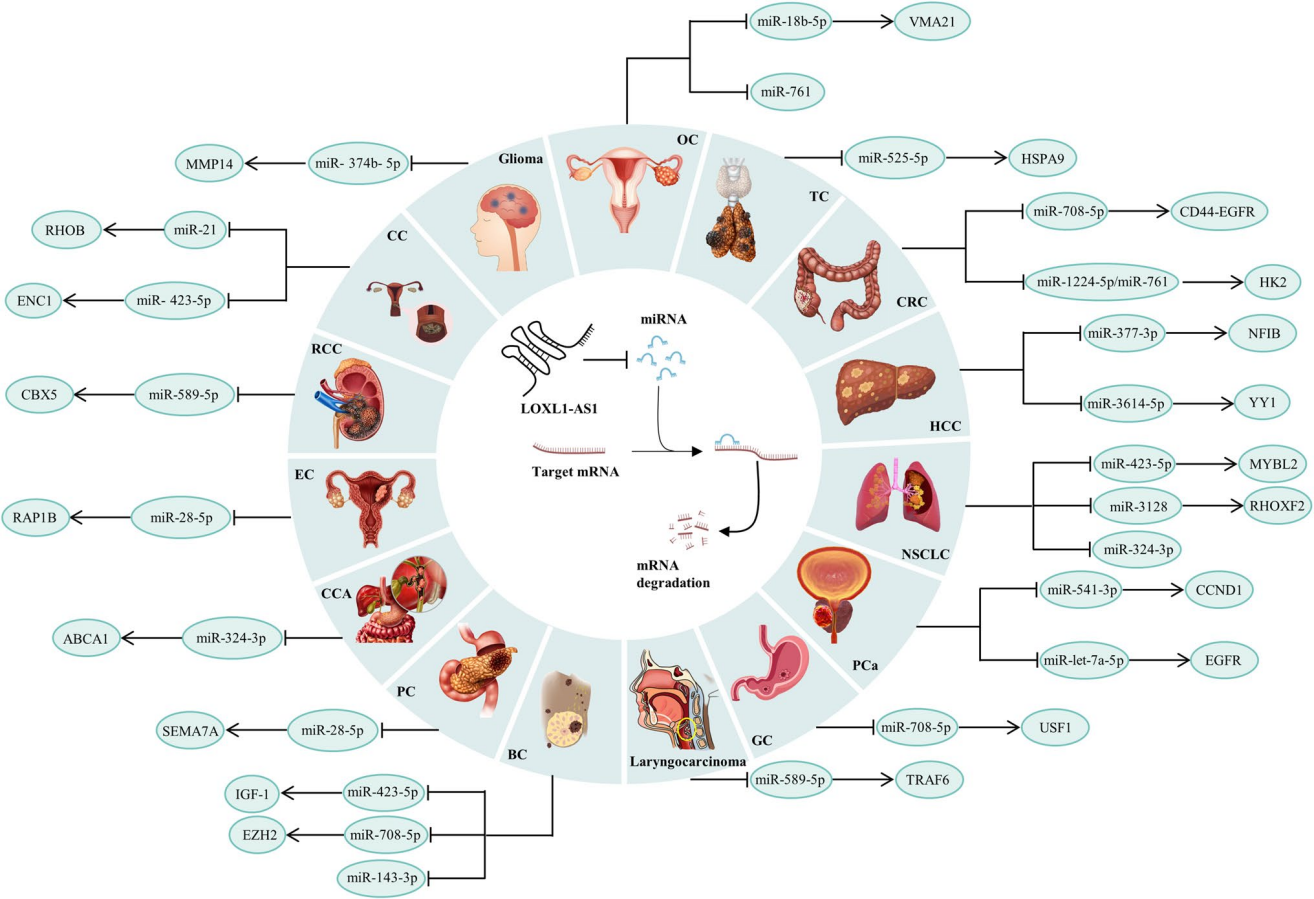
The specific long non-coding RNA (lncRNA)-miRNA–mRNA oncogene regulation mechanism of LOXL1-AS1 in various cancers. The LOXL1-AS1 was significantly overexpressed in tumor tissues. The increased LOXL1-AS1 function as an oncogene and a sponge which targeting miRNAs and activated downstream oncogene pathways

The ceRNA mechanism is an essential method through which lncRNAs can control the expression of other mRNAs and carry out their biological functions. This hypothesis suggests that if both lncRNAs and mRNAs share the same miRNA binding sites, known as miRNA response elements (MREs), they will compete to bind miRNAs and regulate expression [73, 74]. As shown in Fig. 3, LOXL1-AS1 can regulate the expression of its downstream target genes through competitive interaction with miRNA, thus playing a role as an oncogene in different human malignant tumors.

It has been reported that miR-21 [37], miR-761 [48] and miR-324-3p [65] is involved in the carcinogenic activity of LOXL1-AS1, but its downstream targets and their signal pathways or biomolecule interactions need to be further studied. Abnormal expression of hexokinase (HKs) is a feature of glycolysis in cancer cells, while HKs (mainly HK2) is overexpressed in many cancers. LOXL1-AS1 can up-regulate the expression of HK2 and accelerate the progress of CRC by adsorbing miR-1224-5p/miR-761 [20]. Yang et al. [17] confirmed the binding of LOXL1-AS1 to miR-28-5p by luciferase reporter gene detection. Expression analysis showed that the expression of miR-28-5p was up-regulated in shRNA-transfected LOXL1-AS1 cells. They reported that overexpression of miR-28-5p could inhibit the proliferation and migration of PC cells, and further revealed that the downstream molecules of miR-28-5p showed that SEMA7A was the direct target of miR-28-5p [72]. In OC, LOXL1-AS1 acts as an oncogene through the ceRNA network, sponge miR-18b-5p, which promotes the expression of downstream target gene VMA21, thereby enhancing the growth, migration and invasion of OC cells [47]. LOXL1-AS1 also interacts with miR-let-7a-5p to promote the expression of EGFR in PCa. Through the miR-708-5p/USF1 axis, LOXL1-AS1 promotes the progress of GC cells [40]. Silent LOXL1-AS1 inhibits YY1 by binding to miR-3614-5p, thus inhibiting the malignant phenotype of HCC cells [35]. The expression of HSPA9 protein was inhibited by targeting the 3'-untranslated region (UTR) of HSPA9 mRNA. LOXL1-AS1, as a sponge targeting miR-525-5p, promotes the expression of HSPA9, which promotes the growth and invasion and inhibits apoptosis of TC cells [71].

### Involvement in signaling pathways

**Fig. 4:**
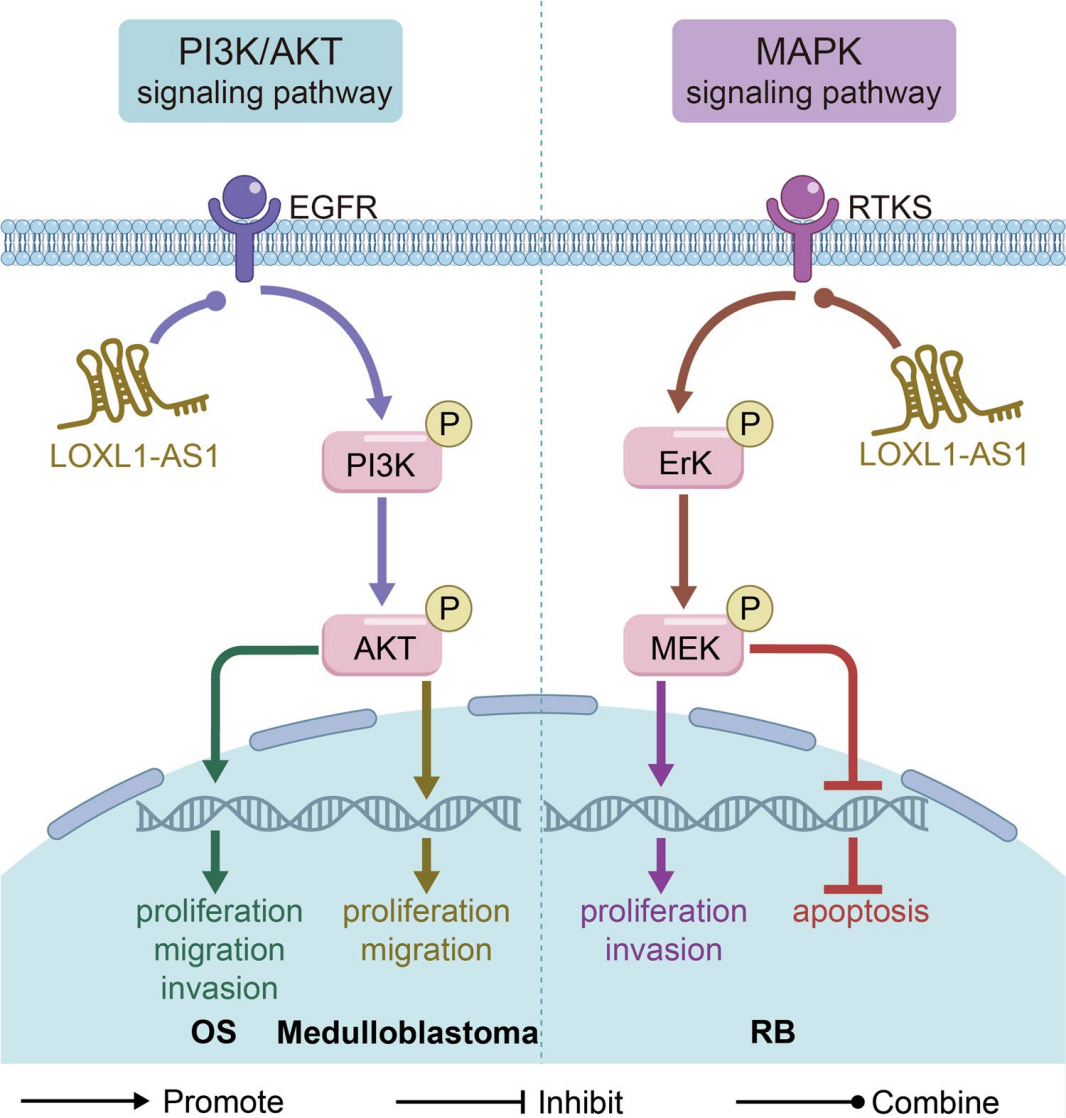
LOXL1-AS1 participates in the PI3K/AKT and MAPK signaling pathways. LOXL1-AS1 regulates the PI3K-AKT signaling pathway by upregulating the phosphorylated proteins of PI3K and AKT to promote the proliferation, migration and invasion of OS [64]. LOXL1-AS1 regulates the PI3K-AKT signaling pathway by upregulating the phosphorylated proteins of PI3K and AKT to promote the proliferation and migration of medulloblastoma [67]. LOXL1-AS1 activates phosphorylated proteins of Erk and MEK protein in MAPK signal pathway to promote proliferation and invasion, and inhibits apoptosis in RB [68]

It has been recognized that MAPK signaling and PI3K/AKT signaling are usually activated in a variety of human cancers to promote their malignant phenotype, which has also been observed in the functional process of LOXL1-AS1 (Fig. 4). Knockdown of LOXL1-AS1 significantly inhibited the proliferation, migration and invasion of OS cells by inhibiting the expression of p-PI3K and p-AKT, but had no effect on PI3K and AKT expression [64]. Gao et al. [67] also confirmed that LOXL1-AS1 promoted the proliferation and metastasis of medulloblastoma by activating the target genes p-PI3K and p-AKT in the PI3K/AKT signaling pathway. In the process of MAPK signal transduction, the phosphorylation of Erk1/2 and MEK1/2 is the key regulatory protein for the activation of the signal pathway, after knocking down LOXL1-AS1 in RB cells, the phosphorylation levels of Erk (p-Erk) and MEK (p-MEK) were significantly decreased, indicating that LOXL1-AS1 positively regulated MAPK signal pathway to promote RB proliferation, invasion and inhibit apoptosis, and the same conclusion was shown in the mouse model of RB xenotransplantation [68].

### Interactions with proteins

LncRNA binds to RNA binding protein (RBP) to form LncRNA-protein complex, which participates in the occurrence and development of cancer by affecting lncRNA and RBP network, subcellular localization and histone modification, which provides a new strategy for the study of corresponding anticancer drugs [75, 76]. DESC1, also known as transmembrane protease serine 11E (TMPRSS11E), is a member of type II transmembrane serine protease (TTSP) family. Recent studies have shown that DESC1 may play a tumor inhibitory role in a variety of malignant tumors, including ESCC [77]. The expression of LOXL1-AS1 in ESCC tissues was significantly higher than that in adjacent non-tumor tissues, and the expression of LOXL1-AS1 was positively correlated with lymph node metastasis in patients with ESCC. Using qRT-PCR to detect the expression of DESC1 in ESCC cells after LOXL1-AS1 knockout, we found that DESC1 was significantly up-regulated. These findings suggest that DESC1 is an important downstream effect factor of LOXL1-AS1 in regulating the progression of ESCC, so LOXL1-AS1 is expected to become a new target for the treatment of ESCC through its interaction with DESC1 protein [70].

### Potential clinical applications of LOXL1-AS1

Although the exact mechanism of LOXL1-AS1 involved in the occurrence and development of human cancer needs to be further studied, LOXL1-AS1 has aroused strong interest and has shown great potential as a biomarker and therapeutic target for diagnosis/prognosis in the future.

### LOXL1-AS1 as a potential diagnostic biomarker

Using modern technology, we detect lncRNAs expression in clinical tissue samples, which makes LOXL1-AS1 to be used as a biomarker for cancer diagnosis. As mentioned above, LOXL1-AS1 is highly expressed in tissue samples from most types of cancer, including EC [17], CRC [22], GC [27], HCC [34], PCa [38], NSCLC [42], OC [47], CC [52], BC [55], gliama [59], OS [64], CCA [65], laryngocarcinoma [66], medulloblastoma [67], RB [68], RCC [69], ESCC [70], TC [71], PC [72]. In normal tissues, LOXL1-AS1 expression is limited. These findings suggest that LOXL1-AS1 may serve as a potential candidate gene for cancer diagnosis.

Evidence that LncRNAs can be released from different types of cancers and detected in the serum or urine of patients [78]. The high stability of LncRNAs in blood and its poor sensitivity to nuclease-mediated degradation make them more reliable than other circulating nucleic acids and may make them more reliable biomarkers of cancer. For example, for GC [79], the HOXA transcript at the distal tip (HOTTIP) in exosomes isolated from the serum may serve as a biomarker with higher diagnostic value than common tumor markers currently used in serum, such as carbohydrate antigen (CA) 19–9, CA 72–4, and carcinoembryonic antigen (CEA). However, the abnormal expression of LOXL1-AS1 reported in most current studies has only been detected at the tissue level. In the future, detection of LOXL1-AS1 expression in serum, plasma, and even exosomes could be used for the diagnosis of these malignancies.

### LOXL1-AS1 as a novel prognostic biomarker

Cancer prognosis monitoring is a key strategy to reduce cancer-related mortality. The expression of LOXL1-AS1 in OC patients was significantly higher than that in normal controls, and its higher expression was strongly correlated with advanced clinical stages. The area under the ROC curve of LOXL1-AS1 is 0.843, the sensitivity is 65.3%, and the specificity is 68.2%. Multivariate analysis confirmed that LOXL1-AS1 was an independent prognostic factor for predicting the prognosis of OC patients. [47]. Chen et al. [64] used Kaplan–Meier method and log-rank test to analyze the correlation between LOXL1-AS1 expression and the overall survival time of patients with OS. The results showed that the overall survival rate of OS patients with high level of LOXL1-AS1 expression was lower than that of OS patients with low level of LOXL1-AS1 expression. Several studies have also shown that the level of LOXL1-AS1 is negatively correlated with the overall survival rate of HCC [36] and glioma [59]. In addition, the higher the expression level of LOXL1-AS1, the later the TNM stage of EC [17], CRC [22], CC [53], BC [55], CCA [65] and ESCC [70], and most of them were accompanied with lymph node metastasis, indicating the poor prognosis. In CRC, higher levels of LOXL1-AS1 can predict liver metastasis of CRC [22]. In summary, dynamic monitoring of LOXL1-AS1 levels may be helpful to assess the severity of malignant tumors and monitor the evolution of cancer. The above studies show the prognostic value of LOXL1-AS1.

### Therapeutic value of LOXL1-AS1 in cancers

LOXL1-AS1 participates in proliferation, apoptosis, migration, invasion, EMT, metastasis and tumorigenesis of different cancers by competing with ceRNA mechanisms. Due to the significant difference in the expression of LOXL1-AS1 between normal and cancer tissues, LOXL1-AS1 gene knockout can slow down the progression of cancer and is a potential new therapy that can be used in the future. Chemotherapy resistance is the main reason for the failure of tumor chemotherapy. The decrease in LOXL1-AS1 gene inhibits the adriamycin resistance of PCa cells through the miR-let-7a-5p/EGFR axis, indicating that LOXL1-AS1 may be a potential therapeutic target for patients with drug-resistant PCa [40]. In the experiment of tumor xenotransplantation in nude mice, over-expression of LOXL1-AS1 showed that PCa tumor was larger and heavier, indicating that the over-expression of LOXL1-AS1 promoted the tumorigenesis of PCa in vivo [40]. In RB cell line, knockdown of LOXL1-AS1 gene inhibits the migration and invasion of RB cells. In the model of RB xenotransplantation, knocking down LOXL1-AS1 can also significantly inhibit tumor growth and promote tumor cell apoptosis, indicating that LOXL1-AS1 is expected to be the basis for clinical treatment of RB [68]. LOXL1-AS1 inhibition using shRNA reduce the motor ability of CRC cells and significantly prevent tumor growth in vivo [23]. As an emerging lncRNA, the research on LOXL1-AS1 is still in pre-clinical stage and more large sample and multicenter studies are needed. We believe that with the deepening of the research, LOXL1-AS1 as the main treatment strategy for cancer will be realized in the future clinical work.

### Critical issues to be solved

Previous studies have described the role and mechanism of LOXL1-AS1 in a variety of cancers, indicating that it has potential clinical value in the diagnosis, treatment and prognosis of cancer. However, due to the current research is very limited, its specific mechanism is still in the early stage of research and many areas still need to be further studied. First, the current studies on LOXL1-AS1 are mainly focused on its downstream regulatory networks or signal pathways, but there are few studies on the potential upstream mechanism of LOXL1-AS1 disorders. The study of upstream regulatory molecules of LOXL1-AS1 may open up a new way for cancer treatment. Secondly, lncRNAs participates in many aspects, such as tumor immunity, metabolism, iron apoptosis, angiogenesis, microenvironment remodeling [80, 81], but the research of LOXL1-AS1 is limited to proliferation, migration, invasion, apoptosis, EMT and so on, so whether LOXL1-AS1 affects other processes still needs to be discussed. Third, lncRNAs regulates gene expression by interacting with DNA, RNA and proteins. However, in most of the published literatures, the regulation of LOXL1-AS1 is limited to the ceRNA mechanism, so it is necessary to further explore other mechanisms of LOXL1-AS1 on the basis of its subcellular localization. In addition, most of the current studies are conducted in preclinical models, and more studies should be conducted at the same time to assess the potential of LOXL1-AS1 in human cancer.

## Discussion

Although ncRNAs has shown therapeutic potential in vivo and in vitro, its limited bioavailability in vivo poses a major challenge to its clinical transformation. In order to overcome this obstacle, there is an urgent need for advanced drug delivery strategies. In order to solve the problems of short half-life, miss effect and low transfection efficiency related to RNA delivery, a variety of ncRNAs vectors and systems have been proposed and widely studied, including several types of nanoparticles, ncRNAs modification and oncolytic adenovirus strategies. While improving the drug delivery system, the safety challenges of ncRNAs therapy also need to be addressed. Meanwhile, deciphering the complex interaction between ncRNA and disease signal pathway and improving the diagnostic ability of existing methods to avoid false positive. Similarly, the proposal of LOXL1-AS1 as a good biomarker for the diagnosis, prognosis and treatment of human cancer is still at the beginning of a long road to clinical application.

As a marker of tumor diagnosis and treatment, lncRNAs have great potential and play an important role in tumor diagnosis, treatment and prognosis. As a new type of lncRNA, LOXL1-AS1 is highly expressed in a variety of cancer samples and cell lines. The abnormal expression of LOXL1-AS1 is closely related to the clinical characteristics of patients, such as tumor size, stage, metastasis and prognosis, indicating that LOXL1-AS1 as a new type of cancer biomarker has great clinical value. LOXL1-AS1 is involved in the regulation of malignant biological processes such as proliferation, migration, invasion, apoptosis and EMT of tumor cells, as well as tumor growth and metastasis. In mechanism, LOXL1-AS1, as ceRNA, affects the biological phenotype of tumor cells by activating miRNAs and regulating target genes and related signal pathways (such as PI3K/AKT signal pathway and MAPK signal pathway).

This article reviews the role and regulatory mechanism of LOXL1-AS1 in many kinds of cancers and introduces the related clinical significance. However, due to the limited sample size, the research of LOXL1-AS1 in cancer is still in the early stage, and there are still a large number of studies to be carried out. It is hoped that this review can provide new insights for clinical development and search for new biomarkers and also provide a reference for future researchers to further study the role of LOXL1-AS1 in cancer.
